# Expanding natural product chemistry resources at the EBI

**DOI:** 10.1186/1758-2946-5-S1-P43

**Published:** 2013-03-22

**Authors:** Janna Hastings, Pablo Conesa, Adriano Dekker, Marcus Ennis, Kenneth Haug, Kalai Jayaseelan, Namrata Kale, Tejasvi Mahendraker, Pablo Moreno, Venkatesh Muthukrishnan, Gareth Owen, Reza Salek, Steve Turner, Christoph Steinbeck

**Affiliations:** 1 Cheminformatics and Metabolism, European Bioinformatics Institute, UK

## 

Natural products are of substantial interest in drug discovery and metabolism research, since they represent molecules that have been shaped by natural selection to be bioactive in ways that are useful for a range of applications including as therapeutics, cosmetics and pesticides. The ChEBI database (http://www.ebi.ac.uk/chebi) and the MetaboLights database (http://www.ebi.ac.uk/metabolights/) aim to offer a comprehensive public resource suite for capturing and describing natural product chemistry. ChEBI has recently added over 2,700 natural products, of which more than 100 have been fully curated. Together with the pre-existing metabolites in ChEBI, the total collection of metabolites (both primary and secondary) is approaching 3,500 entries (October 2012). In addition, we have added the species, strain, and component (e.g tissue type) from which the metabolite has been isolated, linked to the appropriate taxonomies and ontologies, together with supporting citations to the primary literature. The MetaboLights database provides a general-purpose, open-access repository for metabolomics studies, their raw experimental data, and associated metadata [1]. Released in June 2012, the repository includes 15 submitted studies, encompassing 93 protocols for 714 assays over 8 different species. These include species such as H. sapiens, C. elegans, M. musculus and A. thaliana, and techniques such as NMR spectroscopy and mass spectrometry. Finally, we have recently released an open-source, open-data natural product likeness implementation [2], bringing a well-known metric -- useful in compound library screening and lead design - - to a wider community.
